# Successful Pregnancy and Delivery in a Patient with Chronic Myeloid Leukemia while on Dasatinib Therapy

**DOI:** 10.1155/2010/136252

**Published:** 2010-03-07

**Authors:** Monika Conchon, Sabri S. Sanabani, Mariana Serpa, Mafalda M. Y. Novaes, Luciana Nardinelli, Patrícia B. Ferreira, Pedro Enrique Dorliac-Llacer, Israel Bendit

**Affiliations:** ^1^Department of Hematology, Faculty of Medicine, University of São Paulo, SP 05403-000, Brazil; ^2^Fundação Pro-Sangue, Hemocentro de São Paulo, SP 05403-000, Brazil

## Abstract

Here we report the case of an 18-year-old woman with chronic myeloid leukemia (CML) who became pregnant while undergoing treatment with dasatinib. Before pregnancy, she received imatinib mesylate therapy but could not tolerate the treatment. The regimen was then changed to dasatinib at a dose of 70 mg b.i.d. While she was in hematological remission and on dasatinib therapy, she became pregnant. The unplanned pregnancy was identified after the patient had experienced four weeks of amenorrhea. Because the patient elected to continue the pregnancy to term, dasatinib was stopped immediately. Meanwhile, CML hematological relapse occurred and then she was treated with interferon-*α* (IFN-*α*) (9 million IU/day) throughout the pregnancy without a complete hematological response. She successfully gave birth to a male baby at 33 weeks by cesarean section delivery with no sequelae or malformations. Although this experience is limited to a single patient, it provides a useful contribution for counselling patients inadvertently exposed to dasatinib during pregnancy.

## 1. Introduction

CML is a myeloproliferative disorder of blood stem cells [1]. The causative molecular defect is the *bcr-abl *protein, which is encoded by the Philadelphia chromosome (Ph) [2]. This genetic anomaly arises from an exchange of genetic material between chromosomes 9 and 22, which results in the fusion of the breakpoint cluster region (*bcr*) and the Abelson leukemia virus (*abl*) genes. The resulting gene encodes a constitutively active protein kinase that activates a number of proteins involved in cell-cycle regulation that hasten cell division and affect DNA repair. A new class of medications known as TKIs has been developed to selectively inhibit the activity of this abnormal enzyme. Imatinib mesylate is a TKI able to produce long-term suppression of CML in the majority of patients [3]. The drug has become a first-line therapy for newly diagnosed CML patients. As a result, female patients with CML who are of childbearing age and are currently being treated with imatinib now find themselves contemplating reproductive opportunities that would not have otherwise been possible. However, the advantages of this therapy in women of reproductive age are balanced by concerns of imatinib increasing the risk of birth defects. Dasatinib is an oral TKI that binds to active and inactive forms of *bcr-abl *kinase. It is licensed for the treatment of adults with chronic, accelerated, or blast-phase CML with resistance or intolerance to prior therapy, including imatinib. However, the safety profile of inadvertent exposure to this drug in pregnancy is still in question. Here we describe a normal pregnancy outcome in a case of first-trimester exposure to dasatinib in an 18-year-old pregnant woman with CML.

## 2. Case Report

**Figure 1:**
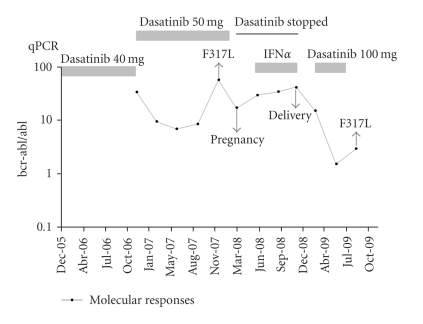
Molecular responses during the entire observation period.

In January 2001, an 18-year-old woman was diagnosed with Ph+ CML in the chronic phase at another hospital. She had been given IFN-*α* 6 × 10^6^ U/day before presenting to our hospital for treatment of CML. Three years after the diagnosis, she was referred to our hospital for further workup. On admission, her physical examination was normal. She was in complete hematological remission, but without any cytogenetic response. Hematological values were: hemoglobin 10.2 g/dL, platelets 447 × 10^9^/L, and white blood cell count 3.69 × 10^9^/L (60% neutrophils, 3% eosinophils, 2% basophils, 20% lymphocytes, 2% monocytes, 0% myeloblasts, 8% promyelocytes, 4% myelocytes, and 1% metamyelocytes). Conventional chromosome banding revealed a 46,XX,t(9;22) karyotype with no other abnormalities. In March 2004, she was started on imatinib at 400 mg daily after giving-written consent. Five months later, she developed grade 4 myelotoxicity evidenced by neutropenia with a neutrophil count of 1 × 10^9^ L. As a result, treatment with imatinib was temporary discontinued. In November 2004, a further attempt with lower-dose imatinib (300 mg daily) combined with granulocyte-colony stimulating factor (G-CSF) again resulted in neutropenia (absolute neutrophil count (ANC) < 100). Because of persistent myelotoxicity, imatinib was permanently withdrawn. In June 2005, after giving an informed consent, she was enrolled in the START Protocol (BMS 180 034) and was slated to receive dasatinib at a dose of 70 mg b.i.d. At that time, the differential peripheral blood count was normal and 85% of the interphase cells were Ph+ by fluorescence in situ hybridization (FISH) assay. The patient was counseled about the teratogenic potential of the agent and was advised to avoid pregnancy while on treatment. After three years of therapy with dasatinib, the number of Ph+ cells decreased from 85% to 25%, but she had severe myelotoxicity (grade 4 neutropenia and grade 4 thrombocytopenia). According to the therapeutic protocols, the dasatinib dose was reduced to 40 mg b.i.d (Figure 1). In July 2008, while she was in hematological remission and on dasatinib therapy, she became pregnant. The unplanned pregnancy was identified during her first trimester of gestation after the patient had experienced four weeks of amenorrhea. This timing indicated that the fetus was inadvertently exposed to the dasatinib. The patient was informed of the potential fetal toxicities of the therapy. After detailed counseling, the patient elected to continue her pregnancy, and dasatinib was stopped immediately. Meanwhile, CML hematological relapse occurred and the F317L mutation in *bcr-abl* was detected. Because of hematological relapse, she was treated with IFN (9 million IU/day) and maintained on the same dose during her pregnancy without complete hematological response. A follow-up with ultrasound scans throughout the course of the pregnancy was unremarkable. In February 2009, she delivered via cesarean section a healthy male baby weighing 2.1 kg with an Apgar score of 9 at ten minutes at gestational week 33. Despite the low birth weight, the infant's growth and development have been normal through 8 months of age without any evidence of congenital malformations. Hydroxyurea therapy at a dose of 1.5 g/day was reinstituted after a few days of delivery and continued for four months. Dasatinib was the only second generation TKI available at that time, and it was reintroduced at the dose of 100 mg/day. Currently, she is in a complete hematologic remission, but failed to achieve cytogenetic or a major molecular response. The patient has been now put on nilotinib therapy.

## 3. Discussion

CML disease in pregnancy is a rare condition, with an annual incidence of 1 per 100,000 pregnancies [4]. The disease during pregnancy has been traditionally managed conservatively by leukapheresis [5, 6], hydroxyurea [7, 8] and interferon [9–11] because it has an initial chronic phase that may not require an immediate therapy and pregnancy *per se *does not affect CML. The therapeutic management of CML in pregnant women with targeted therapies often poses substantial challenges to both patients and their physicians. The main concern is whether to stay on these agents, which carry the risks of birth defects, or stop the medications and risk relapse. The efficacy and safety of molecular target therapies during conception have not been adequately evaluated. This pitfall is attributed mainly to the rarity of CML in young women and the exclusion of pregnant women from active trials. In general, molecular targeted therapies are not recommended during conception because of concerns raised by animal experiments that have suggested an embryotoxic effect of these agents [12–14]. However, it remains uncertain how these effects apply to humans. Currently, much literature exists regarding the evaluation of the outcome of pregnancy while on imatinib and most cases have involved successful pregnancies after the drug is withdrawn. One of the most comprehensive data sets on the effect of imatinib on pregnancy was recently reported by Pye et al. [15]. In Pye et al's. study, imatinib was evaluated in 180 women who were exposed to treatment during pregnancy; outcomes were available for 125 patients. In total, 50% delivered a healthy baby, 28% elected to have a termination and 14% had a miscarriage. The thorough review of the English language literature on women who became pregnant while taking imatinib conducted by Cole et al. [16] identified a total of 217 reported pregnancy events. Of these, 171 carried their pregnancy to term, 24 had spontaneous abortions and 62 had unknown outcomes. Among the 109 pregnancies with the known outcome being that the patient intended to carry their child to term, 36 (33%) had complications, including spontaneous abortion in 24 patients, stillbirth in 1 patient, malformations in 9 patients and low birth weight in 2 patients.

Dasatinib is a multi-targeted kinase inhibitor of *bcr*/*abl *and *Src* kinases that has been developed to overcome intolerance to prior therapy, including the current frontline therapy imatinib, and to suppress the activity of imatinib-resistant *bcr-abl* mutants [17]. The drug is structurally unrelated to imatinib and is able to bind and inhibit both the active and inactive conformations of *abl*, resulting in 100- to 300-fold higher activity than imatinib [17, 18]. Although dasatinib has an excellent efficacy profile, there has been very limited experience with this agent in women with CML who become pregnant while on therapy. To date, only one recent study of the use of dasatinib during early pregnancy has been reported in a scientific meeting [19]. Cortes et al. described the outcomes of pregnancies of 16 patients (8 females and 8 males) who received dasatinib therapy. Of the eight females who conceived while receiving dasatinib, three had therapeutic abortions (two due to patient decision, one for unknown reasons), two had spontaneous abortions (one at eight weeks in a 38-year-old patient [G1P1], one at nine weeks gestation in a 33-year-old [G3P3] and three delivered (one normal, one by cesarean and one unknown). Although none of these women or their neonates experienced serious adverse outcomes, the authors concluded that women of reproductive age who have been started on dasatinib should use effective contraception. There has been only one case report of the use of nilotinib, a second generation TKI, during early pregnancy. Conchon et al. [20] described a 30-year-old mother with CML who became pregnant for the second time while on nilotinib for 7.4 weeks of gestation and delivered a male infant who weighed 3.2 kg at 33 weeks without congenital malformations.

To date, our patient is one of the first reports of a successful pregnancy and delivery of a healthy newborn exposed to dasatinib for approximately eight weeks after conception, during the key period for embryogenesis (3 to 8 weeks of postconception life). We recognize that similar exposure to TKIs may be inadequate for patients with more extensive disease. However, this case and other recent reports [20–22] show that successful outcomes are possible for women with chronic phase CML during pregnancy.

Because conception during treatment is not recommended, couples must be counseled on the risks and benefits of their decision. Although possible serious effects on fetal organs cannot be disregarded in women who become pregnant while receiving therapy, physicians should proceed with caution and closely follow the patients. We believe that the use of TKIs concomitantly with oral contraceptives is still prudent pending more methodologically sound studies involving extensive and adequate clinical data to assess outcomes of TKI-exposed pregnancies and enable the detection of any related fetal defects.
